# A Supra-Physiological Dose of 2-Hydroxyestradiol Impairs Meiotic Progression and Developmental Competence of Mouse Antral Oocytes

**DOI:** 10.3390/jdb13040037

**Published:** 2025-10-15

**Authors:** Valeria Merico, Paola Rebuzzini, Mario Zanoni, Maurizio Zuccotti, Silvia Garagna

**Affiliations:** Laboratory of Biology and Biotechnology of Reproduction, Department of Biology and Biotechnology “Lazzaro Spallanzani”, Via Ferrata 9, University of Pavia, 27100 Pavia, Italy; valeria.merico@unipv.it (V.M.); paola.rebuzzini@unipv.it (P.R.); mario.zanoni@unipv.it (M.Z.); maurizio.zuccotti@unipv.it (M.Z.)

**Keywords:** 2-hydroxyestradiol, estrogen metabolites, oocyte maturation, meiotic progression, cytoskeletal organisation, embryo development, microtubule-organizing centers, F-actin cap

## Abstract

Estrogen metabolites (EMs) play a local regulatory role in mammalian ovarian function. Among them, 2-hydroxyestradiol (2-OHE2) exerts dose-dependent effects on reproductive physiology, supporting either normal ovarian processes or contributing to pathological conditions. Specifically, 2-OHE2 modulates ovarian vasculature and progesterone biosynthesis, and at 1–10 nM concentrations, it enhances in vitro developmental competence and blastocyst quality in mouse oocytes. Conversely, doses below 1 nM show no appreciable effects, suggesting the existence of a biological activity threshold. However, the impact of supra-physiological concentrations remains largely unexplored. In this study, we investigated the effects of increasing 2-OHE2 doses (0.05, 0.50, and 5.00 µM) on oocyte meiotic progression and quality. Exposure to 0.50 and 5.00 µM significantly impaired oocyte maturation, while only the highest dose notably reduced the percentage of embryos developing to the blastocyst stage. Morphometric analysis during the GV-to-MII transition revealed altered first polar body morphology, defective asymmetric division, and disruptions in cytoskeletal organization, including enlarged meiotic spindles, increased F-actin cap angles, and aberrant microtubule-organizing centers distribution. These structural alterations were paralleled by distinct changes in cytoplasmic movement velocity patterns observed through time-lapse imaging during meiotic resumption. Together, these findings demonstrate that supra-physiological exposure to 2-OHE2 compromises oocyte maturation and developmental competence by perturbing key cytoskeletal dynamics and cellular architecture necessary for successful meiosis and early embryogenesis.

## 1. Introduction

Estrogen metabolites (EMs) are water-soluble products of estrogen (E2) hydroxylation. 2-Hydroxyestradiol (2-OHE2) and 4-hydroxyestradiol (4-OHE2) are the main metabolites, which can further be converted into methoxy-derivatives, 2-methoxyestradiol (2Me-OHE2) and 4-methoxyestradiol (4Me-OHE2), respectively [1]. EMs were initially thought to be inactive or have low binding affinity for E2 receptors [2,3], but a local regulatory role in the mammalian ovarian function has been more recently clearly demonstrated [4,5,6,7,8]. For example, 2-OHE2, with its pro-angiogenic activity, regulates human ovarian vasculature, promoting blood vessel formation during follicle selection, rupture, and corpus luteum (CL) development [5,6,8]. On the contrary, 2Me-OHE2 exerts an anti-angiogenic role in follicular neo angiogenesis and in CL physiological regression [5,6,8].

The range of EMs is essential for the regulation of reproductive processes, either activating or inhibiting them, supporting physiological regularity or accompanying pathological conditions. Throughout the menstrual cycle, the variation levels of EMs are finely tuned. For example, 2-OHE2 increases 2.5-fold around ovulation, reaching 93.62 nM in urine, compared to 62.42 nM in the pre- and post-ovulatory phases [9,10]. Altered levels of 2-OHE2 have been linked to reproductive disorders. In polycystic ovary syndrome (PCOS), 2-OHE2 levels are significantly reduced in both urine and follicular fluid compared to ovulatory women [6,11]. In contrast, women with endometriosis show elevated levels of 2-OHE2 in the eutopic endometrium, which may contribute to the pathophysiology of this condition [12].

In vitro studies have shown that 2-OHE2 stimulates the production of the vascular endothelial growth factor in human granulosa cells [4,5]. This EM also influences progesterone biosynthesis in small follicles: it stimulates porcine granulosa cells to produce progesterone [13] and promotes progesterone accumulation in rat luteal cells [14]. In the mouse, the presence of 1 nM or 10 nM 2-OHE2 during in vitro maturation of cumulus-oocyte complexes (COCs) enhances oocyte developmental competence, increasing the rate of preimplantation development and improving blastocyst quality [15]. While doses below 1 nM do not exhibit significant effects on oocyte competence or embryo quality, highlighting a threshold requirement for biological activity [15], the consequences of exposure to higher concentrations remain to be elucidated.

Beyond 2-OHE2, other EMs have also been shown to negatively impact gamete and embryo development when present at supra-physiological concentrations. In vitro studies demonstrated that the acquisition of full developmental competence in both bovine [16] and mouse [17] oocytes is impaired by high doses of 2Me-OHE2 (2.5–7.5 µM). In bovine, after oocyte fertilization, cleaved embryos developed up to the morula stage, but blastocyst formation was prevented by developmental arrest and aneuploidy associated with disrupted nuclear maturation [16]. Chromosome segregation errors and nondisjunction were also observed in mouse oocytes exposed to 3.75 µM 2Me-OHE2 [17]. Further support that high EMs exposure induces oxidative stress and DNA damage was obtained in complementary studies using normal breast epithelial cells (MCF-10A) [18,19,20].

These findings indicate that nuclear maturation, chromosomal stability, and embryo development can be impaired by altered levels of EMs, suggesting the importance of defining their effects in oocyte biology.

Circulating concentrations of 2-OHE2 are normally low (≈20–200 pg/mL, ≈0.07–0.7 nM) [12,21], but local dysregulation of estrogen metabolism can lead to abnormal accumulation in pathologies such as PCOS or endometriosis. In PCOS, the metabolic balance of catechol-estrogens is shifted by altered hydroxylation and reduced detoxification capacity [6,22]. In endometriosis, aberrant expression of CYP1A1/1B1 enhances catechol-estrogen formation within the eutopic endometrium and peritoneal fluid, increasing oxidative stress [12]. Moreover, environmental endocrine disruptors may saturate metabolic pathways and favor catechol-estrogen accumulation [21]. Since a finely tuned redox microenvironment modulates oocyte maturation and early embryonic development; supra-physiological concentrations of EMs—though unlikely systemic—could likely occur in ovarian follicles or ectopic endometrial lesions. Under these conditions, increased 2-OHE2 may impair meiotic progression and preimplantation development, thus justifying the experimental exploration of micromolar concentrations as a model of pathological exposure.

In this study, mouse ovarian antral COCs were subjected to in vitro maturation (IVM) in the presence of 2-OHE2 at supra-physiological concentrations (0.05, 0.5, and 5.0 µM). We then assessed (i) the oocytes’ capacity to resume meiosis and support embryonic development up to the blastocyst stage following fertilization, and (ii) key cellular events associated with meiotic progression. Furthermore, considering that such cytological processes can be monitored as global cytoplasmic movements using bright-field time-lapse microscopy [23], we analyzed cytoplasmic movement velocity (CMV) profiles [24] to evaluate whether 2-OHE2 exposure alters the temporal dynamics of the germinal vesicle (GV) to metaphase II (MII) transition.

## 2. Materials and Methods

### 2.1. Animals and Reagents

Four- or five-week-old female and five-month-old male CD1 mice (Charles River Laboratories, Como, Italy) were kept in an animal house under controlled conditions (22 °C, 60% air moisture and 12:12 light:dark photoperiod). Animal manipulation and investigations were conducted in accordance with the guiding principles of European (2010/63/UE) and Italian (Dlvo 26/2014) laws and the experimental protocols approved by the University Ethical Committee and by the Italian Istituto Superiore di Sanità (protocol numbers 909/2018-PR and 919/2023-PR).

2-OHE2 powder (Merck, Milano, Italy) was dissolved in dimethyl sulfoxide (DMSO; Merck) to a stock solution of 5 mM. The stock solution was maintained at −20 °C. At least four hours before use, the stock solution was diluted in the oocyte maturation medium (see next paragraph) to final concentrations of 0.05, 0.50, or 5.00 µM 2-OHE2.

### 2.2. Cumulus-Oocyte-Complexes Isolation and In Vitro Maturation

CD1 females were injected with 10 U of pregnant mare’s serum gonadotropin (PMSG; Intervet, Segrate, Italy), 48 h before their sacrifice. Isolated ovaries were placed in HEPES-buffered α-MEM W/GLUTAMAX-I (ThermoFisher Scientific, Monza, Italy) supplemented with 6 mg/mL HEPES, 5% fetal bovine serum (FBS; ThermoFisher Scientific), 0.23 mM sodium pyruvate (Merck), 1 mg/mL fetuin (Merck), 100 U/mL penicillin and 75 µg/mL streptomycin (ThermoFisher Scientific).

Isolation of COCs was carried out by puncturing the ovarian surface with a sterile 26G needle (Merck) for their release. COCs with fully-grown antral oocytes completely surrounded by at least 3–4 layers of follicle cells were collected and washed in fresh drops of IVM medium containing: α-MEM W/GLUTAMAX-I (ThermoFisher Scientific-Gibco, Segrate, Italy) supplemented with 5% FBS, 0.23 mM sodium pyruvate (Merck), 1 mg/mL fetuin (Merck), 100 U/mL penicillin (ThermoFisher Scientific-Gibco), 75 µg/mL streptomycin (ThermoFisher Scientific-Gibco), 50 mU/mL follicle-stimulating hormone (FSH; Merck-Calbiochem, Milano, Italy) and 10 ng/mL epidermal growth factor (EGF; Merck). Then, COCs were divided into three groups and cultured in: (i) IVM-only medium, (ii) the presence of 0.001, 0.01 and 0.1% DMSO, the 2-OHE2 vehicle, (iii) the presence of 0.05, 0.50 or 5.00 µM 2-OHE2. Then the three groups of COCs were in vitro matured for 7 h up to metaphase I (MI) or for 15 h up metaphase II (MII) in 5% CO_2_ at 37 °C.

The highest (0.1%) DMSO concentration used did not show adverse effects on IVM and preimplantation embryo development (Table S1), confirming previously reported results [15,25]. For this reason, the DMSO results were subsequently used as the control for comparison with the 2-OHE2 results.

### 2.3. Morphology and Size of PB-I

The morphology and size of the first polar body (PB-I) were assessed as previously described [15]. In brief, CC-free MII oocytes were positioned under an inverted Olympus IX71 microscope (Olympus Italia S.r.l., Segrate, Italy) equipped with a JVC KY-F58 3-CCD camera (Olympus Italia S.r.l) and a micromanipulator to obtain the maximum PB-I diameter in both frontal and lateral orientations. Images were acquired at 10× magnification, and three lines representing the maximum diameters were drawn using CellSens Dimension 1.4.1 software (Figure S1). The half of three diameters were used to calculate the PB-I volume, approximating its shape to an ellipsoid.

### 2.4. In Vitro Fertilization

MII oocytes were inseminated with 1.8 × 10^6^/mL capacitated sperm at 37 °C and 5% CO_2_ for 2 h as previously described [18]. Presumptive zygotes (as determined by the presence of a second polar body, PB-II) were transferred in M16 medium (Merck) (2 μL/oocyte) supplemented with 0.4% bovine serum albumin (BSA; Merck), 2 mM glutamine (ThermoFisher Scientific-Gibco), 5 mM taurine (Merck) and 0.23 mM pyruvate (Merck) for preimplantation development. The presence of pronuclei in presumptive zygotes was evaluated 6 h post-insemination using an inverted microscope. Embryonic development rate was evaluated at 24 (2-cell stage), 48 (4-cell) and 96 (blastocyst) h post-insemination.

### 2.5. Immunofluorescence and Analyses of Oocyte Geometrical Parameters

Detection of α-tubulin, γ-tubulin and F-actin in MI and MII oocytes was performed as previously reported [15]. Briefly, oocytes were fixed and permeabilized in 4% paraformaldehyde, diluted in a Microtubule Stabilization Buffer, supplemented with 1% Triton X-100 (Merck) for 35 min at 37 °C in gentle agitation. After washing in Wash Solution (WS) containing 1× PBS (Merck), 2% FBS, 1% BSA (Merck), 1% powder milk (Merck), 0.2% 36 glycine (Merck) and 0.5% Tween20 (Merck), gametes were incubated with the primary antibodies diluted in WS (anti-α-tubulin 1:1000, cat. n. T5168; anti-γ-tubulin 1:500, cat. n. T5326; Merck), at 37 °C for 1 h in gentle agitation and then with an AlexaFluor 488 conjugated goat secondary anti-mouse IgG (ThermoFisher Scientific-Life Technologies, Segrate, Italy) diluted 1:2000 in WS together with Phalloidin-TRITC (cat. n. P1951, Merck) diluted 1:1000. Oocytes’ nuclei were counterstained with 0.2 μg/mL DAPI (Merck) for 10 min at room temperature and mounted in Vectashield Antifade Mounting Medium (cat. n. H-1000; Vector Laboratories-DBA, Segrate, Italy).

Samples were analyzed using a Leica TCS SP8 confocal microscope (Leica, Parma, Italy) equipped with a white laser and the AOBS system (Leica). Stacks were obtained with axial distances of 0.3 μm. Images were processed with ImageJ 1.54p version (http://imagej.nih.gov/ij/; accessed on 1 January 2021).

The extension and intensity of the cortical F-actin cap, the meiotic spindle shape and area and the microtubule-organization centers (MTOCs) localization were analyzed as reported in Reference [15].

MII oocytes stained with DAPI were also evaluated for chromosome misalignment. One or more chromosomes were considered misaligned when detached or when showing a lagging phenotype. Lagging chromosomes are defined as those chromosomes that fail to clear the central spindle region after 15 h IVM.

### 2.6. Time-Lapse Recording and Analyses

Denuded oocytes were cultured in 2 μL drops of IVM medium (4 oocytes/drop) in the presence or absence of 5 µM 2-OHE2 onto a 3.5 cm glass-bottom dish (WillCoWells, Amsterdam, The Netherlands), covered with mineral oil (Merck), at 37 °C and 5% CO2 in the incubator of a BioStation IM (Nikon, Moncalieri (TO), Italy). Time-lapse images of the GV-to-MII transition were acquired with a 20× objective, under bright-field light (intensity set to 39), with an exposure time of 1/20 s, and a gain of 1.41 [24], using the BioStation IM-Q software (version 2.0) at 5 min intervals over a total 15 h (180 frames). This time interval was sufficient to capture all major events of mouse oocyte maturation during the GV-to-MII transition, which typically occur at intervals of at least 30 min [23,24,26].

The Cell_PIV software (MATPIV v6.6.1), developed and kindly provided by Dr. Shane Windsor [27,28], was used to detect cytoplasmic movement velocity (CMV) during the GV-to-MII transition in both CTR and 5 µM 2-OHE2 exposed oocytes. Cytoplasmic movements were measured by cross-correlation between patterns of pixels and those of the subsequent frame. Velocity vectors represent the movement of a pattern of pixel, in the cytoplasm, between sequential frames: different lengths and colors correspond to different magnitudes of movement. Cell_PIV software uses a series of MATLAB tools (V.25.1) (PIV, PROCESS and VIEWER Tools) and works with grey scale images of a single oocyte. PIV Tool specifies the location of the cells on an image and calculates the velocity data; Process Tool calculates the mean velocity magnitude in the center of the region of interest and outputs data files and graphs (.csv, .tif, respectively); Viewer Tool allows the view of PIV Tool results and exports videos, images and data files (.avi, .tif, .csv/.xls, respectively). At the end of the analysis, the Cell_PIV software provided the raw data of the cytoplasmic movement, used as parametric values to perform analysis for comparison. The calculated mean velocities are expressed in nm/min.

### 2.7. Statistical Analysis

Statistical analyses were carried out using the Sigma Stat 3.5 and Prism 10 software. Data, obtained from at least five independent experiments, were analyzed by the Student’s t test or by the one-way ANOVA, followed by the Fisher LSD Method (post hoc test). The Mann–Whitney test or the Kruskal–Wallis test together with the Dunn’s multiple range test were used for the statistical analysis of nonparametric data. Data were expressed as the mean ± SEM (standard error of the mean) or mean ± SD (standard deviation). The statistical analysis of the absolute frequencies was performed using a Fisher’s exact test. Differences were considered significant for *p* values ≤ 0.05.

## 3. Results

### 3.1. The Presence of 5.00 µM 2-OHE2 During IVM Reduces Both Oocyte Meiotic and Developmental Competence

Following 15 h of IVM with 0.1% DMSO (control, CTR), the 2-OHE2 vehicle, 92% of fully-grown antral oocytes reached the MII stage (Table 1; Figure S2A), confirming earlier reports [15,21]. A very similar maturation rate (*p* > 0.05) was obtained in the presence of 0.05 µM 2-OHE2. Instead, at 0.50 or 5.00 µM 2-OHE2 exposure, the frequency of MII oocytes was significantly lower, 87% (*p* < 0.001) or 78% (*p* < 0.001), respectively. In the latter group, more than 20% (*p* < 0.01) of oocytes were either blocked at the GV or germinal vesicle breakdown (GVBD) stages or were pyknotic/fragmented (Table 1 and Figure S2B,C).

The presence of 2-OHE2 in the culture medium at concentrations of 0.50 and 5.00 µM significantly reduced the frequency of antral oocytes capable of completing meiosis and reaching the MII stage, suggesting an impact of both concentrations on the correct progression of the first meiotic division.

Next, to assess MII developmental competence, matured oocytes were in vitro inseminated, and embryos were cultured to the blastocyst stage.

Following fertilization, 62% of CTR MII reached the 2-cell stage and of these about 31% completed preimplantation development up to the blastocyst stage (Table 2; Figure S2D,E), a frequency expected when COCs are in vitro grown [15,29,30]. When compared to CTR samples, 0.05 or 0.50 µM 2-OHE2-exposed MII oocytes showed no significant difference (*p* ≥ 0.05) in their developmental efficiency, at all embryo stages (Table 2). Instead, only 37% of 5.00 µM 2-OHE2-exposed oocytes reached the 2-cell stage and, of these, only 5% developed to blastocyst (*p* < 0.001) (Table 2; Figure S2F,G). To determine whether the reduction in developmental rate in the 5.00 µM group was also related to a decrease in fertilization rates, we assessed the presence of two pronuclei in presumptive zygotes 6 h post-insemination using an inverted microscope. No significant differences were observed between the groups (73.77 ± 5.26% vs. 64.52 ± 9.12% in CTR and 5.00 µM 2-OHE2-exposed oocytes, respectively; *p* = 0.085).

Overall, these results indicate that the presence of 0.50 and 5.00 µM 2-OHE2 in the culture medium of fully-grown oocytes disrupts meiotic progression to MII and, at the highest dose, impacts on preimplantation embryonic development.

### 3.2. The Presence of 5.00 µM 2-OHE2 Alters Cellular Features of Cell Division and Meiotic Spindle Organization in MI and MII Oocytes

The 5.00 µM dose of 2-OHE2 disrupts both meiotic progression and preimplantation embryonic development. Thus, only this dose was considered in subsequent experiments. We evaluated the perturbation of key cellular events associated with meiotic progression, including: the progressive clustering of MTOCs, crucial in spindle formation during meiosis [31], the shape of the meiotic spindle and the alignment of chromosomes, vital indicators of oocyte quality reflecting fertilization success and embryo development rates [32,33], the amplitude of the F-actin cap at the site of PB-I extrusion [34], and the volume of the PB-I, a defining feature of the asymmetry of female meiosis [35].

#### 3.2.1. MTOC Dynamics

**Figure 1:**
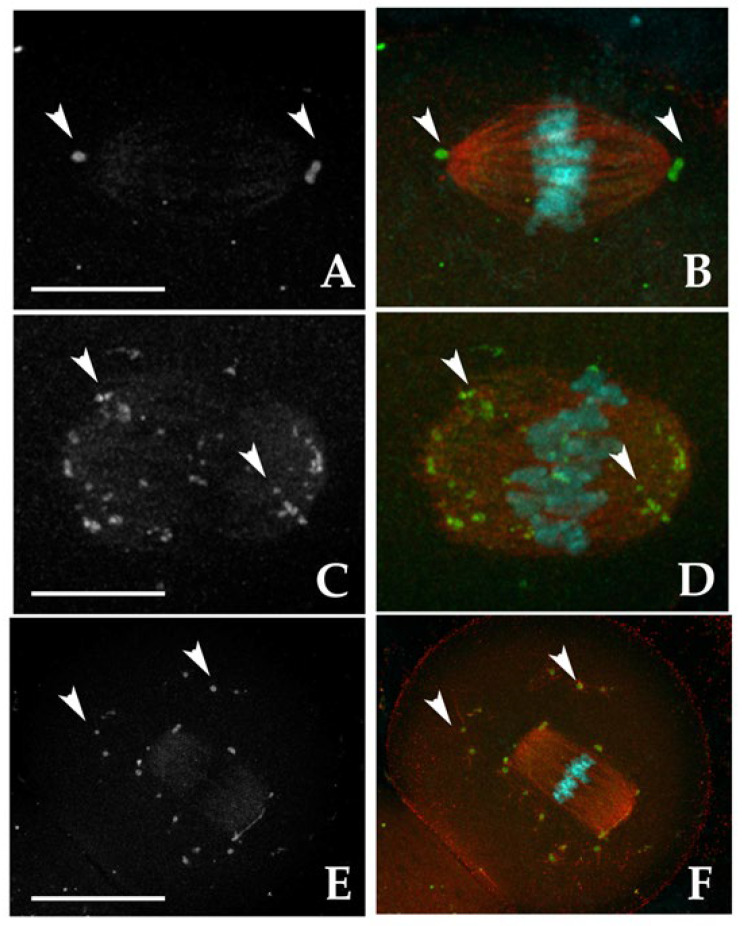
Representative fluorescent images showing the localization of microtubule-organizing centers (MTOCs, white arrowheads) in MII oocytes immunostained with γ-tubulin antibody (grayscale in **A**,**C**,**E**; green in **B**,**D**,**F**). The meiotic spindle was visualized with α-tubulin antibody (red in **B**,**D**,**F**), and chromosomes were counterstained with DAPI (cyan). Class I: MTOCs clustered at the two spindle poles (**A**,**B**). Class II: MTOCs distributed along spindle fibers (**C**,**D**). Class III: MTOCs dispersed throughout the cytoplasm (**E**,**F**). Scale bars: 10 µm (**A**–**D**); 30 µm (**E**,**F**).

For this analysis, MTOCs were categorized into two main types based on their roles in spindle formation (Figure 1). The first one is represented by polar MTOCs (pMTOCs) that contribute directly to spindle pole formation [36,37] and are further divided into two classes. Class I MTOCs (Figure 1A,B) are initially located in the perinuclear region [38]. They decondense and redistribute into smaller foci before GVBD [37,39], eventually clustering to form the spindle poles [36,40,41] whereas Class II MTOCs (Figure 1C,D) disperse throughout the cytoplasm during GVBD and later migrate from the periphery to the oocyte’s center to contribute to spindle pole formation [36,37]. The second main type, cytoplasmic MTOCs (cMTOCs), were classified as Class III (Figure 1E,F). These MTOCs remain in cytoplasm without contributing to spindle formation, and their function remains unclear [31].

In CTR MI oocytes, all analyzed samples exhibited pMTOCs. Specifically, 64.1% displayed Class I MTOCs at the spindle poles, 12.8% exhibited dispersed γ-tubulin signals along spindle fibers (Class II), and 23.1% showed a combination of Class I and II. Additionally, 82.1% of MI oocytes had Class III MTOCs, with 79.5% presenting a few clustered γ-tubulin spots (≤30), while 2.6% contained numerous (>30) smaller, dispersed spots (Table S2). The remaining 17.9% lacked cMTOCs. Exposure to 2-OHE2 significantly reduced the proportion of MI oocytes with exclusively Class I MTOCs to 14.3% (*p* < 0.001), while increasing the prevalence of Class II MTOCs to 53.6% (*p* < 0.001). In 28.6% of cases, both Class I and II were present. Class III MTOCs remained common (78.2%, *p* ≥ 0.05), but 25% of exposed oocytes contained >30 widely dispersed γ-tubulin signals (*p* = 0.005 vs. CTR) (Table S2).

In CTR MII oocytes, the majority (97%) displayed pMTOCs, with 60.6% exhibiting Class I MTOCs, 15.2% Class II, and 21.2% a mix of both classes (Figure 1A–D). Class III MTOCs were observed in 69.7% of cases (Figure 1E,F). Following 2-OHE2 exposure, the proportion of oocytes with Class I MTOCs dropped to 24.2% (*p* = 0.005), while Class II increased to 45.2% (*p* = 0.002); 22.6% exhibited both classes. Class III MTOCs were present in 90.4% of exposed oocytes (*p* = 0.04), with 32.3% (*p* = 0.039) containing >30 small, highly dispersed γ-tubulin signals. The percentage of MII oocytes lacking Class III MTOCs decreased to 9.7% (*p* = 0.04) (Table S2).

#### 3.2.2. Shape of the Meiotic Spindle

The meiotic spindles of both CTR and 2-OHE2-exposed MI and MII oocytes were visualized using anti-α-tubulin immunolabelling.

**Figure 2:**
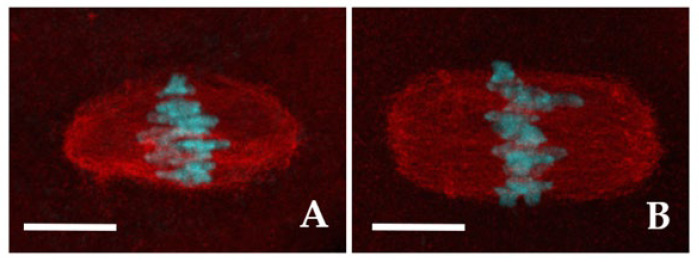
Meiotic spindle morphology. Representative fluorescent images of MI spindles with barrel- (**A**) or rectangular-shape (**B**). The spindle fibers were labelled with α-tubulin (red) and chromosomes were counterstained with DAPI (cyan). Bars: 5 µm.

In the vast majority of CTR (93.6%, *n* = 47) and in 71.9% (*n* = 55) of 2-OHE2-exposed MI oocytes the spindle fibers displayed a well-organized spindle morphology. Among these, 72.7% of CTR oocytes exhibited a barrel-like spindle shape (Figure 2A), compared to 56.4% in the 2-OHE2-exposed group (*p* ≥ 0.05) (Table 3). While the mean spindle area did not differ significantly between groups (*p* = 0.099), the barrel-like spindle pole width was significantly larger in 2-OHE2-exposed oocytes (*p* = 0.021) (Table 3). In MI oocytes with a rectangular-like spindle configuration (Figure 2B), 2-OHE2 exposure significantly increased both the mean equatorial spindle width (*p* < 0.001) and the mean area, reaching 398.8 ± 31.6 µm^2^ (*p* = 0.007) (Table 3). In the remaining 6.4% of CTR MI oocytes and 29.1% of 2-OHE2-exposed MI oocytes (*p* < 0.05), only dispersed α-tubulin signals surrounding misaligned chromosomes were observed (Figure S3A,D).

Similar trends were observed in MII oocytes. Among CTR MII oocytes, 61.7% displayed a barrel-like spindle shape, compared to 55.8% in the 2-OHE2-exposed group (*p* ≥ 0.05) (Table 3). Treatment with 2-OHE2 significantly increased both the equatorial width and area of barrel- and rectangular-like spindles. Additionally, in rectangular-like spindles, the pole width was significantly larger than in CTR oocytes (*p* = 0.001) (Table 3).

Although most oocytes in both the CTR and treated groups displayed a typical barrel-shaped spindle, the higher proportion of oocytes with abnormal spindle morphologies in the treated group suggested a potential alteration in chromosome alignment. To examine this, we compared the percentage of unaligned chromosomes across the different spindle types and between the two groups. In CTR MII oocytes, the frequency of chromosome misalignment did not differ significantly (*p* = 0.324) between those with a barrel-shaped spindle (4.8%) and those with a rectangular spindle (14.3%), although a trend toward higher misalignment was observed in the rectangular group. In contrast, in 2-OHE2–treated MII oocytes, the frequency of misaligned chromosomes was significantly higher in rectangular spindles (52.6%) compared with barrel-shaped spindles (16.0%) (*p* = 0.009). Notably, when comparing between CTR and treated groups, chromosome misalignment was significantly increased in 2-OHE2–treated oocytes with a rectangular spindle (*p* = 0.033), but not in those with a barrel-shaped spindle (*p* = 0.223).

#### 3.2.3. F-Actin Cap Angle Extension

The F-actin cap was visualized using phalloidin staining and measured in a total of *n* = 90 CTR and *n* = 143 2-OHE2-exposed MI oocytes, as well as in *n* = 96 CTR and *n* = 86 2-OHE2-exposed MII oocytes.

**Figure 3:**
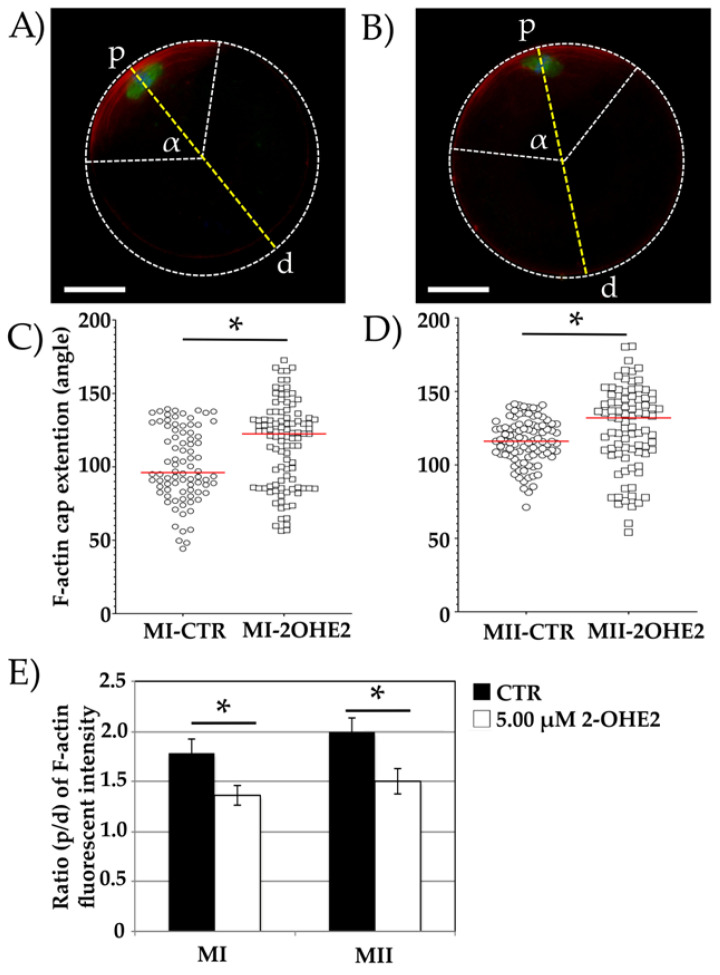
Representative images of the cortical F-actin cap (red channel) in MI or MII oocytes from control (CTR) (**A**) and 5.00 µM 2-OHE2-exposed (**B**) groups. The green channel (**A**,**B**) represents α-tubulin. α is the angle of the circular sector defined by the two white lines extending from the oocyte centroid to the endpoints of the F-actin cap arc. Points “p” and “d” represent opposite ends of a dotted line (yellow) drawn through the oocyte’s centroid, intersecting the MI (**A**) or MII (**B**) plate. Bar: 25 µm. Plots showing the F-actin cap angle (α, dotted white lines) extension in MI (**C**) and MII (**D**) oocytes. Red lines indicate median values *; *p* < 0.05. (**E**) Histograms showing the relative fluorescence intensity of the F-actin cap in MI and MII oocytes, expressed as the p/d ratio (intensity at point “p” divided by that at point “d”) * *p* < 0.05.

A well-defined cortical F-actin cap was observed in 92.9% (*n* = 84) of MI CTR oocytes and 75.5% (*n* = 101) of 2-OHE2-exposed MI oocytes (Figure 3A,B). The remaining oocytes displayed an F-actin signal distributed across the entire oolemma without a distinct cap formation. A significantly higher proportion of 2-OHE2-exposed MI oocytes (24.5%, *n* = 42) compared to CTR oocytes (7.1%, *n* = 6) exhibited this abnormal F-actin distribution (*p* < 0.05; Figure S3B,D). Due to the absence of a distinct F-actin cap, these oocytes were excluded from further analysis.

In oocytes exhibiting proper cortical F-actin cap distribution, the angle of the circular sector formed by the F-actin arc was measured to assess its amplitude. In CTR MI oocytes, the mean angle was 101.1°, with a maximum value of 139.4° (Figure 3A,C). In contrast, MI oocytes exposed to 2-OHE2 exhibited a significantly increased median angle of 115.7° (*p* < 0.001), with 19% of oocytes showing values between 139.4° and 172.7° (Figure 3B,C).

After 15 h of IVM, all MII oocytes extruded the PB-I displayed and an F-actin cap. The mean angle in CTR MII oocytes was 116.0°, whereas exposure to 2-OHE2 significantly increased this value by approximately 10° (*p* = 0.008) (Figure 3D).

Furthermore, fluorescence intensity analysis revealed a significantly higher (*p* < 0.05) F-actin cap signal in both MI and MII CTR oocytes compared to those matured in the presence of 2-OHE2 (Figure 3E).

#### 3.2.4. Volume of the First Polar Body

**Figure 4:**
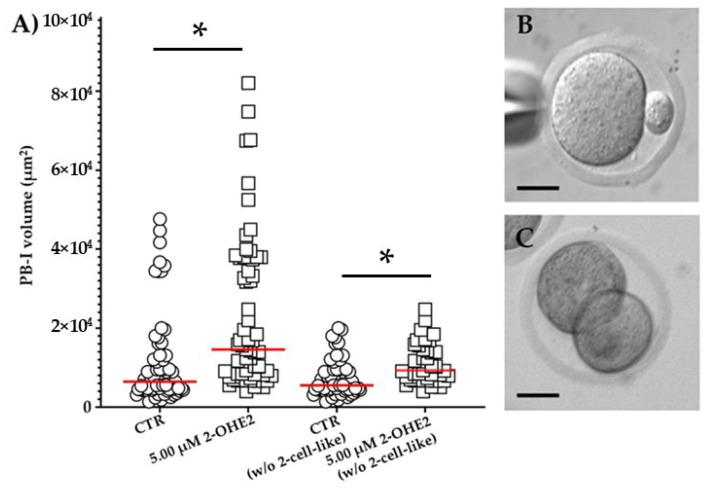
(**A**) Distribution of PB-I volume for control (CTR) and 2-OHE2-exposed oocytes. The analysis includes (CTR or 5.00 μM 2-OHE2) or excludes (CTR or 5.00 μM 2-OHE2 excluding 2-cell-like embryos) oocytes from the count. Red bar represents the median value. * *p* ≤ 0.001. Examples of MII oocyte with a PB-I derived after an asymmetric (**B**) or symmetric, 2-cell-like configuration, division (**C**); bar: 30 µm.

In MII CTR oocytes, the mean PB-I volume was 1.2 × 10^4^ ± 1.5 × 10^3^ µm^3^ (Figure 4A,B). Following exposure to 5.00 µM 2-OHE2, the mean volume significantly (*p* ≤ 0.001) increased up to 2.3 × 10^4^ ± 2.4 × 10^3^ µm^3^ (Figure 4A). Moreover, in 14% of CTR group, oocytes display a 2-cell-like configuration (having a PB-I volume > 3 × 10^4^ µm^3^) (Figure 4C), while the 5.00 µM 2-OHE2-treated oocytes show a marked increase, reaching 34% (*p* = 0.006) of oocytes having a 2-cell-like configuration, indicative of a symmetrical division. Furthermore, it is interesting to note that even following the exclusion of 2-cell-like oocytes from the comparison, the PB-I remained significantly (*p* = 0.0032) larger in the 5.00 µM 2-OHE2-treated group than in the CTR group (Figure 4A).

### 3.3. Cytoplasmic Movement Velocity Profiles During the GV-to-MII Transition

The previous observations indicated that 2-OHE2 influences crucial events during the resumption of meiosis. In earlier studies by coupling time-lapse bright-field microscopy with image analysis based on particle image velocimetry (PIV), we and others [24,27,28] demonstrated that cytological events occurring during the GV-to-MII transition can be visualized as global cytoplasmic movements. To assess whether 2-OHE2 influences the timing of cellular events during the GV-to-MII transition we recorded the CMV profiles [24].

**Figure 5:**
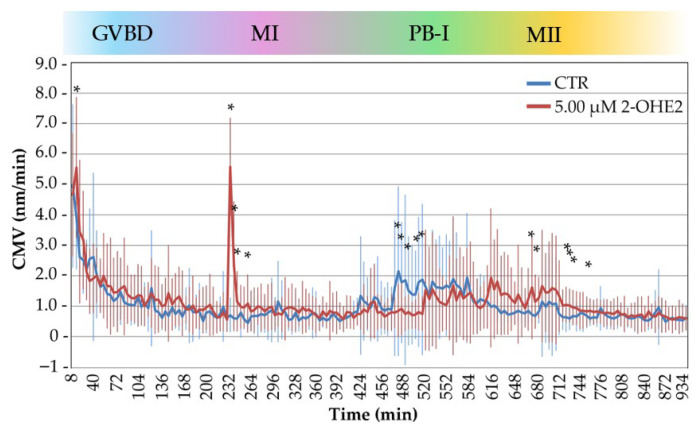
Cytoplasmic movements velocity (CMV) profiles of control (CTR) and 2-OHE2-exposed oocytes recorded over a 15 h period during the GV-to-MII transition. The color bar indicates the progression of oocyte maturation phases: GVBD (germinal vesicle breakdown), MI (metaphase I), PB-I (polar body I), and MII (metaphase II). * *p* < 0.05.

Figure 5 summarizes the CMV profiles of 32 CTR and 30 2-OHE2-exposed oocytes, aligning time-lapse measurements with critical cytological events during the GV-to-MII transition. While both groups displayed broadly similar CMV trends over time, statistical analysis revealed significant differences during four key cellular events.

In the initial phase of GVBD (0–20 min), corresponding to the onset of nuclear envelope disassembly, 2-OHE2-exposed oocytes displayed a significant increase in CMV (5.56 ± 0.46 nm/min in the 2-OHE2 group and 3.91 ± 0.30 nm/min in the CTR group, respectively; *p* = 0.0018). In this group, 40% of the oocytes showed an increase of up to twofold higher than the CTR. This early surge in cytoplasmic activity may reflect premature signaling events or altered meiotic entry mechanisms, consistent with previous observations on early oocyte activation dynamics [24,42].

During the spindle assembly and chromosome alignment phase (225–265 min), when chromosomes adopt a rosette-like configuration and the MI spindle forms, oocytes exposed to 2-OHE2 showed a significant increase in CMV (*p* = 0.005). This activity peaked at 235 min, where these oocytes exhibited a mean CMV that was eightfold higher (5.57 ± 1.37 nm/min) than the CTR group (0.72 ± 0.10 nm/min). Notably, this period coincides with spindle formation and preparation for cortical migration [23,43,44].

During PB-I extrusion (465–510 min), CTR oocytes showed an increase in CMV, reaching up to twofold higher than the treated oocytes, which corresponds to the beginning of PB-I extrusion. In contrast, 2-OHE2-exposed oocytes exhibited a significant reduction in CMV (*p* ≤ 0.05), suggesting that this altered cytoplasmic activity may reflect impaired meiotic progression [29] and aligning with a notable delay in PB-I extrusion in the treated group (645 min vs. 552 min in CTR; *p* ≤ 0.001). Specifically, only 20% of oocytes exposed to 2-OHE2 extruded PB-I by 510 min.

Finally, following the formation of the MII spindle (665–765 min), CTR oocytes showed a gradual decrease in CMV, indicating stabilization of cytoplasmic dynamics. Conversely, 2-OHE2-exposed oocytes exhibited a sustained CMV elevation (*p* ≤ 0.05) reaching up to twofold higher than CTR, implying continued intracellular activity or delayed cytoplasmic reorganization. During this period, 45% of treated oocytes extruded PB-I. After 765 min, CMV levels in 2-OHE2-exposed oocytes declined and converged with those of the CTR group, suggesting a partial recovery or eventual alignment in cytoplasmic behavior at later stages.

## 4. Discussion

Our findings demonstrate that exposure to supra-physiological levels of 2-OHE2 during the GV-to-MII transition adversely affects oocyte maturation and developmental competence. Specifically, oocytes treated with 0.50 and 5.00 µM 2-OHE2 exhibited significantly reduced maturation rates, with increased arrest at the GV and GVBD stages, and a lower percentage reaching the MII stage. Following fertilization, developmental competence was markedly compromised only in oocytes exposed to the highest concentration, with just 37% progressing to the 2-cell stage and 5% forming blastocysts. These results indicate that exposure to 5.00 µM 2-OHE2 not only disrupts meiosis but also exerts long-lasting defects that impair early embryonic development.

In human biological fluids, physiological concentrations of 2-OHE2 are typically in the nanomolar range (≈0.07–0.7 nM in serum or follicular fluid, corresponding to ≈20–200 pg/mL) [15,31]. In contrast, the concentrations used in this study (0.05–5.0 µM) exceed these levels by several orders of magnitude. While systemic occurrence of such levels is unlikely, local accumulation of estrogen metabolites may occur in pathological or exposure-related contexts. For example, in endometriosis, increased CYP1A1/1B1 activity favors catechol estrogen accumulation in the eutopic endometrium and peritoneal fluid with local concentrations exceeding systemic levels [12]. In PCOS, altered estrogen hydroxylation disrupts the balance of EMs in follicular fluid. Consequently, anti-angiogenic EMs levels increase up to 2-fold in antral follicles of anovulatory PCOS women compared to ovulatory women [6]. Environmental endocrine disruptors and pharmacological estrogens may also overload detoxification pathways, promoting local enrichment of reactive estrogen metabolites [45,46,47]. Within this framework, testing micromolar concentrations provides translational insight into how abnormal estrogen metabolism could compromise oocyte competence.

The observed impairment was accompanied by profound cytoskeletal disruptions at both the MI and MII stages. The cytological defects observed in our model included enlarged F-actin cap, altered spindle dimensions at both MI and MII and delayed chromosome alignment at the MII stage. In MI oocytes, this increase was specific to spindles exhibiting a rectangular configuration, while in MII oocytes, both barrel-shaped and rectangular spindles showed greater width at the poles and equator. These changes are consistent with impaired spindle migration and loss of asymmetric division, as previously shown when actin dynamics are perturbed [15,43,44,48,49,50,51,52,53]. In our study, although 78% of oocytes exposed to 5.0 µM 2-OHE2 reached MII, 34% of them displayed a two-cell-like morphology suggestive of defective cytokinesis. This phenotype aligns with models in which cortical actin and myosin-II coordinate the pushing and pulling forces required for spindle migration and anchoring at the cortex [43,51,52,54]. Recent work has further shown that precise regulation of cortical actin is required to maintain asymmetric spindle positioning in MII oocytes, thereby preventing symmetric cleavage [55,56]. Our observations suggest that the expanded F-actin cap induced by 2-OHE2 disrupts this anchoring process, providing a mechanistic explanation for the altered cytokinesis we report.

Notably, both spindle size and polar body dimensions have been proposed as non-invasive markers of oocyte competence, as they reflect proper cytoskeletal organization—key to successful meiotic progression and embryo development. Deviations in these structural parameters, particularly in MII spindles, are known to correlate with reduced fertilization rates and impaired blastocyst formation, with both undersized and oversized spindles falling outside the functional window of competence [15,31,49,57,58]. The morphological anomalies observed in our model thus further substantiate the detrimental impact of 2-OHE2 on oocyte quality.

In addition to actin-related defects, exposure to 2-OHE2 altered MTOC dynamics. At the highest dose, we observed an increased number of cytoplasmic MTOCs with aberrant clustering and mis localization along the spindle and at its poles. Around 45% of oocytes displayed rectangular spindles instead of the typical barrel-shaped morphology. In MI oocytes, spindle enlargement was limited to rectangular spindles, whereas in MII oocytes it was observed in both spindle morphologies, indicating broader cytoskeletal disruption. Our model demonstrated that 2-OHE2 modifies the meiotic spindle phenotype, leading to an increased proportion of oocytes with a rectangular spindle. Notably, in these oocytes more than 50% of metaphase plates exhibited chromosome misalignment. This is particularly relevant, as proper MTOC clustering and distribution are essential for accurate spindle assembly and chromosome alignment, and their disruption has been associated with abnormal chromosome segregation and reduced oocyte competence [59]. The aberrant MTOCs behavior observed in our study supports the hypothesis that 2-OHE2 interferes with cytoskeletal reorganization, contributing to meiotic defects [31]. Furthermore, bright-field time-lapse imaging revealed perturbations in CMV during critical meiotic events, including GVBD, spindle assembly at MI and MII, and PB-I extrusion. These alterations indicate that 2-OHE2 affects not only static cytoskeletal structures but also the dynamic cytoplasmic processes that drive meiotic progression.

2-OHE2 exerts detrimental effects on oocyte maturation through mechanisms that are likely multicausal, potentially involving both receptor-mediated and non-receptor pathways. Although 2-OHE2 displays relatively low affinity for estrogen receptors [7], an ER-dependent signaling contribution cannot be excluded. Several lines of evidence suggest that non-receptor mechanisms may have a prevalent role. One possible pathway is linked to the intrinsic redox properties of catechol estrogens: 2-OHE2, at high concentrations, undergoes redox cycling, leading to the generation of reactive oxygen species (ROS) and oxidative DNA damage [60,61]. It is well known that oxidative stress destabilizes the meiotic spindle, delays chromosome alignment, and compromises developmental competence [49,62]. In addition, ROS production has been associated with activation of JNK phosphorylation, which localizes to spindle structures in mouse oocytes and may represent a signaling link between oxidative imbalance and cytoskeletal dysfunction [63].

A second possible mechanism involves the metabolic conversion of 2-OHE2 into its methoxylated derivative, 2-MeOHE2. This latter has been shown to be a microtubule-disrupting agent capable of binding tubulin and destabilizing spindle integrity [64]. In accordance with this, it has been shown that supra-physiological concentrations of 2-MeOHE2 impair oocyte nuclear maturation and increase aneuploidy in both cattle and mice [16,17]. Therefore, at the micromolar concentrations tested in this study, the deleterious effects observed could arise from the combined action of 2-OHE2 itself and its conversion to 2-MeOHE2.

Finally, our findings point to a direct impact of 2-OHE2 on the MTOCs, which abnormally cluster and mis localize in treated oocytes, providing strong evidence that cytoskeletal reorganization is compromised at a structural level, leading to defective spindle assembly and chromosome segregation [59,65]. Together, these mechanisms highlight the complexity of 2-OHE2 action on the oocyte and suggest that both oxidative stress and cytoskeletal destabilization converge to impair meiotic progression and developmental potential.

## 5. Conclusions

We reported that supra-physiological 2-OHE2 concentrations compromise oocyte quality by disrupting both static (F-actin cap, spindle structure, MTOC clustering) and dynamic (CMV) cytoskeletal processes, critical for meiotic progression and early development. From a clinical perspective, these results suggest that abnormal intraovarian accumulation of estrogen metabolites or through environmental exposures, represents a potential risk factor for impaired fertility. Importantly, cytoskeletal markers such as spindle size and polar body dimensions are increasingly recognized as non-invasive predictors [66] of oocyte competence, strengthening the translational relevance of our findings.

## Figures and Tables

**Table 1:** Rate of fully grown antral oocytes progression into metaphase II (MII) when cultured in the absence (CTR) or presence of 2-OHE2. IVM, in vitro maturation; COC, cumulus oocyte complex; GV, germinal vesicle; GVBD, germinal vesicle breakdown; SEM, standard error of the mean; * *p* < 0.01; ** *p* < 0.001.

Treatment	% ± SEM (Number) of Oocytes During IVM			
	COC	Blocked at GV or GVBD	Fragmented or Pyknotic	MII
CTR	100 (416)	6.13 ± 0.67 (25)	1.46 ± 0.51 (6)	92.41 ± 0.61 (385)
2-OHE2 (µM)				
0.05	100 (155)	5.58 ± 1.61 (10)	1.46 ± 0.60 (3)	92.98 ± 1.04 (166)
0.50	100 (315)	8.34 ± 0.65 (13)	4.64 ± 0.79 * (7)	87.04 ± 0.79 ** (135)
5.00	100 (330)	11.14 ± 1.42 * (39)	10.70 ± 0.96 ** (33)	78.16 ± 1.40 ** (258)

**Table 2:** Rate of in vitro preimplantation embryonic development of metaphase II (MII) oocytes derived from cumulus oocyte complexes previously matured in the absence or presence of 2-OHE2. SEM, standard error of the mean; * *p* < 0.001.

Treatment	% ± SEM (Number) of Preimplantation Embryos			
	Inseminated MII	2-cell	4-cell **	Blastocyst **
CTR	100 (223)	61.71 ± 3.86 (130)	53.82 ± 6.02 (69)	31.41 ± 1.60 (41)
2-OHE2 (µM)				
0.05	100 (144)	66.66 ± 3.18 (93)	53.96 ± 2.72 (50)	36.62 ± 2.08 (33)
0.50	100 (133)	64.72 ± 4.76 (83)	45.50 ± 7.21 (36)	28.88 ± 2.06 (24)
5.00	100 (118)	36.64 ± 2.83 * (42)	38.58 ± 5.69 (15)	5.00 ± 5.00 * (2)

** The developmental rate was calculated based on the number of 2-cell embryos (set at 100%).

**Table 3:** Frequency (%) and geometrical features measurements (mean ± SEM) of barrel- and rectangular-like spindles in control (CTR) or 5.00 µM 2-OHE2-exposed MI or MII oocytes. * *p* = 0.021; ** *p* ≤ 0.007.

	MI		MII	
	CTR	2-OHE2	CTR	2-OHE2
Barrel spindle				
% (N)	72.7 (32)	56.4 (22)	61.7 (21)	55.8 (25)
Equatorial width (µm)	13.8 ± 0.9	14.9 ± 0.9	11.1 ± 0.3	14.0 ± 0.5 **
Pole width (µm)	7.0 ± 0.3	8.4 ± 0.5 *	4.5 ± 0.2	4.8 ± 0.3
Area (µm^2^ ± SEM)	363.2 ± 15.5	423.2 ± 38.8	143.0 ± 4.4	255.4 ± 15.9 **
Rectangular spindle				
% (N)	27.3 (12)	43.6 (17)	38.3 (13)	44.2 (19)
Equatorial width (µm)	11.6 ± 0.9	14.7 ± 1.2	11.0 ± 0.2	13.5 ± 0.7 **
Pole width (µm)	6.6 ± 0.5	11.2 ± 0.7 **	6.0 ± 0.3	7.8 ± 0.4 **
Area (µm^2^ ± SEM)	283.7 ± 13.2	398.8 ± 31.6 **	144.2 ± 16.5	278.6 ± 45.7 **

## Data Availability

The original contributions presented in this study are included in the article/Supplementary Materials. Further inquiries can be directed to the corresponding author.

## Supplementary Materials

The following supporting information can be downloaded at: https://www.mdpi.com/article/10.3390/jdb13040037/s1, Table S1: Rate mean ± S.D. (number) of in vitro maturation and preimplantation embryonic development of cumulus-oocyte-complexes (COCs) cultured in absence or presence of 0.1% DMSO (vehicle); Figure S1: Lateral and frontal views of a first polar body (PB-I); Figure S2: Representative images of in vitro matured MII oocytes cultured in absence (CTR) or presence of 5.00 µM 2-OHE2 and of 2-cell (D,F) and blastocysts (E,G) embryos derived from in vitro fertilized CTR (D,E) or 5.00 µM 2-OHE2-exposed (E,G) MII oocytes; Table S2: Absolute percentage of Class I, II and III microtubule-organization centers (MTOCs) in control (CTR) and 2-OHE2-exposed MI or MII oocytes (5.00 µM); Figure S3: Fluorescence microscopy images showing the absence of a spindle fiber and of an F-actin cap in an oocyte after 6 h of in vitro culture.
