# Supplementary material for: A Supra-Physiological Dose of 2-Hydroxyestradiol Impairs Meiotic Progression and Developmental Competence of Mouse Antral Oocytes
Source: J Dev Biol. 2025 Oct 15;13(4):37. doi: 10.3390/jdb13040037 (PMC12551066; doi:10.3390/jdb13040037)
Supplement: Supplementary file 1 [file jdb-13-00037-s001.zip › jdb-3805375-supplementary.pdf]

Table S1. Rate of *in vitro* maturation and preimplantation embryonic development of cumulus-oocyte-complexes (COCs) cultured in the absence or presence of 0.1% DMSO. Data were analyzed using a *t*-test. No significant differences were observed between the two groups.

Colture medium	% ± S.D. (number) of oocytes and preimplantation embryos at different stage of maturation or development							
	COC	Blocked at GV or GVBD	Fragmented or pyknotic	MII	Inseminated MII	2-cell	4-cell	Blastocyst*
without DMSO	100 (149)	4.8 ± 2.0 (6)	2.2 ± 2.7 (3)	93.3 ± 3.4 (140)	100 (120)	63.9 ± 10.7 (77)	36.8 ± 4.5 (44)	31.3 ± 6.2 (23)
with DMSO	100 (190)	6.3 ± 2.0 (12)	2.7 ± 1.6 (5)	91.1 ± 1.1 (173)	100 (158)	62.0 ± 6.6 (97)	32.5 ± 5.0 (51)	28.2 ± 4.9 (27)

(*): The developmental rate was calculated based on the number of 2-cell embryos (100%).

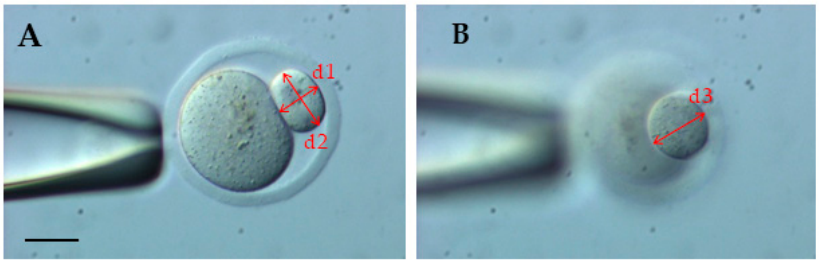

Figure S1: MII oocytes showing (A) lateral and (B) frontal views of the first polar body (PB-I). The three lines (d1, d2, and d3) represent the diameters from which three radii were calculated to determine the PB-I volume, approximating its shape to an ellipsoid. Scale bar: 20 µm.

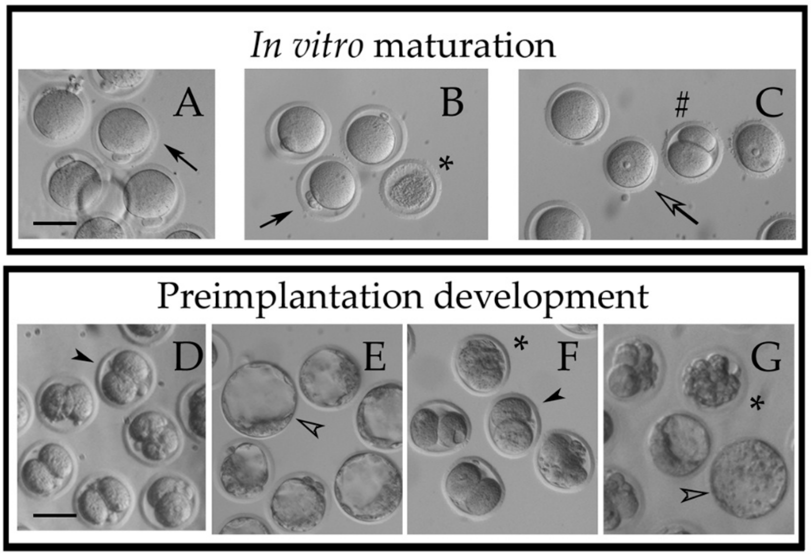

Figure S2. (A-C) Representative images of *in vitro* matured MII oocytes cultured in the absence (A) or presence (B-C) of 5.00 µM 2-OHE2. MII oocytes, arrows; oocytes blocked at the GV stage, empty arrow; degenerated oocyte, asterisk; 2-cell-like MII oocyte, hash symbol. Bar = 80 µm.

(D-G) Representative images of 2-cell (D,F) and blastocysts (E,G) embryos. CTR (D,E); 5.00 µM 2-OHE2-exposed (E,G) MII oocytes. 2-cell embryo, arrowheads; blastocysts, empty arrowheads; degenerated embryo, asterisks. Bar = 100 µm.

Table S2. Absolute percentage (number) of Class I, II and III microtubule organization centers (MTOCs) in control (CTR) and 5.00 µM 2-OHE2-exposed MI or MII oocytes. pMTOCs, polar MTOCs; cMTOCs, cytoplasmic MTOCs. * $p < 0.05$

**MTOCs**			**MI**		**MII**	
			**CTR**	**2-OHE2**	**CTR**	**2-OHE2**
pMTOCs	CLASS I		64.1 (25)	14.3 (4)*	60.6 (20)	24.2 (8)*
	CLASS II		12.8 (5)	53.6 (16)	15.2 (5)	51.6 (16)
	CLASSES I+II		23.1 (9)	28.6 (8)	21.2 (7)	22.6 (7)
cMTOCs	CLASS III	≤ 30 spots	79.5 (31)	53.2 (19)	60.6 (20)	58.1 (18)
		> 30 spots	2.6 (1)	25.0 (7)*	9.1 (3)	32.3 (10)*

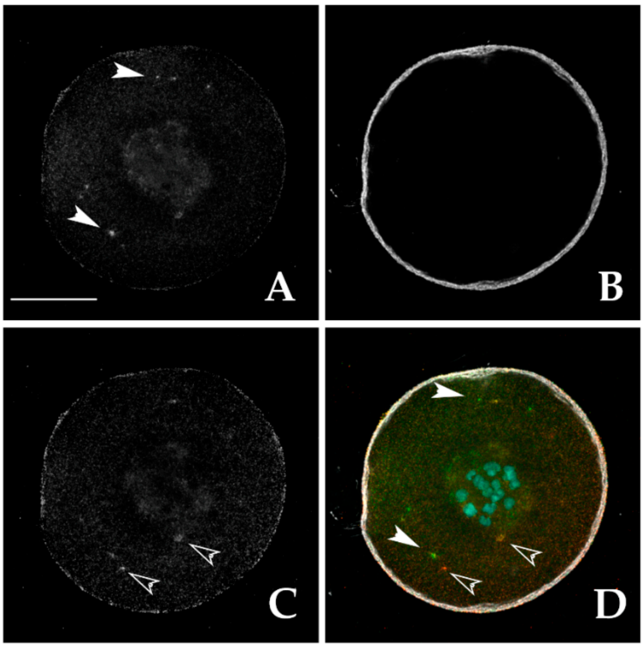

Figure S3. Fluorescence microscopy images showing the absence of a spindle fiber and of an F-actin cap in an oocyte after 6 hours of in vitro culture. This example illustrates a deviation from the typical organization. (A) α-tubulin spots (white arrowheads); (B) F-actin localisation; (C) γ-tubulin spots (empty arrowheads). (D) Merged images; α-tubulin (green); F-actin (grey); γ-tubulin (red). Chromosomes were counterstained with DAPI (cyan). Scale bar: 15 µm.
